# Non-Coding RNAs in Muscle Dystrophies

**DOI:** 10.3390/ijms141019681

**Published:** 2013-09-30

**Authors:** Daniela Erriquez, Giovanni Perini, Alessandra Ferlini

**Affiliations:** 1 Department of Pharmacy and Biotechnology, University of Bologna, Bologna 40126, Italy; E-Mail: daniela.erriquez@hotmail.it; 2 Health Sciences and Technologies–Interdepartmental Center for Industrial Research, University of Bologna, Bologna 40064, Italy; 3 Section of Microbiology and Medical Genetics, Department of Medical Sciences, University of Ferrara, Ferrara 44100, Italy

**Keywords:** microRNAs (miRNAs), long non-coding RNAs (lncRNAs), Duchenne muscular dystrophy (DMD), Becker muscular dystrophy (BMD), Myotonic dystrophies (DM1 and DM2), Facioscapulohumeral dystrophy (FSHD)

## Abstract

ncRNAs are the most recently identified class of regulatory RNAs with vital functions in gene expression regulation and cell development. Among the variety of roles they play, their involvement in human diseases has opened new avenues of research towards the discovery and development of novel therapeutic approaches. Important data come from the field of hereditary muscle dystrophies, like Duchenne muscle dystrophy and Myotonic dystrophies, rare diseases affecting 1 in 7000–15,000 newborns and is characterized by severe to mild muscle weakness associated with cardiac involvement. Novel therapeutic approaches are now ongoing for these diseases, also based on splicing modulation. In this review we provide an overview about ncRNAs and their behavior in muscular dystrophy and explore their links with diagnosis, prognosis and treatments, highlighting the role of regulatory RNAs in these pathologies.

## 1. Introduction

Transcription of the eukaryotic genome yields only 1%–2% of protein coding transcripts and the remainder is classified as non-coding RNAs (ncRNAs). In other words, non-coding RNAs are the main output of the global transcription process, highlighting the idea that such an intense cellular effort cannot be just simple noise. Rather, it is reasonable to speculate that this underscored transcriptome possesses specific vital functions [1–3].

In general, non-coding RNAs are divided into structural and regulatory RNAs. The first ones include ribosomal, transfer, small nuclear and small nucleolar RNAs (rRNAs, tRNAs, snRNAs and snoRNAs respectively), which have been deeply characterized at the functional level. The second ones are a very broad class of RNAs whose main categorization essentially relies on their length.

Small ncRNAs are defined as transcripts shorter than 200 nucleotides. The most functionally characterized are microRNAs (miRNAs), piwi-interacting RNAs (piRNAs) and small interfering RNAs (siRNAs), which are critical for the assembly and the activity of the RNA interference machinery.

RNAs longer than 200 nucleotides are named long non-coding RNAs (lncRNAs) and are a very heterogeneous group of molecules. Because there is not an official way to classify them, they can be placed in one or more categories depending on their genome localization and/or on their orientation (sense, antisense, bidirectional, intronic or intergenic lncRNAs) [4,5].

In the past years, several reports have increased our knowledge about additional levels of regulation of many physiological processes that are mediated by ncRNAs. Even more interesting, these flexible molecules have been found to be dysregulated in many pathological human disorders.

In this review, we will focus on RNAs involved in human skeletal muscle dystrophies. There is a continuous flow of new scientific reports that underpin functional links between ncRNAs and skeletal muscle biology, suggesting that these molecules can play a crucial function both in physiological muscle development and in pathological muscle disorders.

Muscular dystrophies (MDs) are strictly inherited conditions recognized as a common pathogenic mechanism of disruption/impairment of the muscle cell membrane (sarcolemma) which causes a cascade of pathogenic events, including: inflammation, cell necrosis and cell death with progressive fibrosis replacing the muscle mass. MDs represent diseases of extraordinary interest both in medical genetics and biology. The high translational value of research about MDs has recently driven scientific findings toward precise genetic diagnoses as well as novel therapies [6–9].

There are more than 30 different types of inherited dystrophies that are characterized by muscle wasting and weakness of variable distribution and severity, manifesting at any age from birth to middle years, resulting in mild to severe disability and even short life expectancy in the worse cases. Clinical and pathological features are generally the parameters to classify the most common type of MDs. The broad spectrum of MDs arises from many different genetic mutations that reflect defects not only in structural proteins, but also in signaling molecules and enzymes. Dystrophin was the first mutant structural protein shown to cause MD. Mutations in the dystrophin gene lead to two more common type of dystrophy: the severe Duchenne muscular dystrophy (DMD OMIM 300677) due to out-of-frame mutations, and the milder Becker muscular dystrophy (BMD OMIM 300376) associated with in-frame mutations. Some “exceptions to the reading frame rule” are associated with intermediate phenotypes. The genetic causes of the highly heterogeneous Limb Girdle Muscular Dystrophies (LGMDs) reside in many genes (such as α, β, γ, δ, and ɛ sarcoglycans) encoding for structural proteins that are part of the complex sarcolemma network and deeply involved, together with dystrophin, in force transduction [10,11].

There are other muscular dystrophies, such as the Facioscapulohumeral muscular dystrophy (FSHD OMIM 158900) and Myotonic dystrophies (DM1 and DM2, see below), that are due to mutations in genes with a main regulatory function. FSHD is due to deletions in non-coding RNA which cause modification of the chromatin assembly in the 4q34 chromosomal region; Myotonic dystrophies (DMs) are related to trinucleotide (DM1) and tetranucleotide (DM2) repeat expansions that produce toxic mutant mRNA with subsequent interference of RNA-splicing mechanisms [12,13].

Many lines of evidence reveal that aberrant expression levels of non-coding RNAs can result in novel types of defects that cause remarkable changes in processes such as mRNA maturation, translation, signaling pathways or gene regulation. To date, it is clear that there is involvement of several miRNAs in the muscular dystrophies, on the contrary, very little is known about the role of long ncRNAs [14].

In this review we try to recapitulate the emerging studies about this intriguing category of molecules, summarizing what is known in muscle, both in physiological and in pathological contexts; new insights are revealing that they are important players in processes such as cellular lineage commitment, growth and differentiation of skeletal muscle. Since muscle differentiation and regeneration are key features that require to be considered when designing novel therapies, addressing the role of ncRNAs in MDs is of high clinical relevance.

## 2. Muscle-Specific and Ubiquitously Expressed miRNAs in Skeletal Muscle

microRNAs control the stability and/or the translational efficiency of target messenger RNAs, thus causing post-transcriptional gene silencing. Mammalian miRNAs are transcribed as long primary transcripts (pri-miRNAs) and encode one or more miRNAs. Pri-miRNAs are processed by RNase III Drosha in the nucleus to generate stem-loop structures of ~70 nucleotides (pre-miRNAs) and then exported to the cytoplasm where they are further processed by RNase Dicer to yield ~22 bp mature miRNAs. A mature miRNA, incorporated into the RNA-induced silencing complex (RISC), anneals to the 3′ UTRs of its target mRNAs by its complementary strand, thus causing post-transcriptional gene silencing via translational repression or mRNA degradation. In the last years new paradigms of miRNA biogenesis are also emerging in which the processing of miRNA does not require all steps mentioned above [15].

Vertebrate skeletal muscle is derived from the somites, the first metameric structures in mammalian embryos, that progressively subdivide into embryonic compartments, thus giving rise to dermomyotome and subsequently to myotome to produce differentiated muscular tissue. The process of generating muscle—myogenesis—is highly complex and requires a broad spectrum of signaling molecules, either during embryonic development and in postnatal life, that converges on specific transcription and chromatin-remodeling factors, as well as on regulatory RNAs, to activate gene and microRNA expression program [16,17].

The fate of myogenic precursor cells is first determined by paired-homeodomain transcription factors, Pax3/Pax7, followed by regulation of highly conserved MyoD (also named MyoD1, myogenic differentiation 1), Myf5 (myogenic factor 5), MyoG (myogenin), and MRF4 factors, expressed in the skeletal muscle lineage and therefore referred as myogenic regulatory factors (MRFs). The MRFs differ in the timing and the stages of myogenesis, reflecting their different roles during muscle cell commitment and differentiation. MyoD and Myf5 are both considered markers of terminal commitment to muscle fate. Myf5 is the first MRF expressed during the formation of the myotome, followed by expression of MyoD. Specifically, in the majority of muscle progenitors, MyoD functions downstream from Pax3 and Pax7 in the genetic hierarchy of myogenic regulators, whereas Myf5, depending on the context, can also act in parallel with the Pax transcription factors [18–20]. Instead, MyoG and MRF4 act subsequently to specify the immature muscle cells (myoblasts) for terminal differentiation. Myoblasts exit from cell cycle after a defined proliferation time, to become terminally differentiated myocytes [21,22]. Muscle-specific genes such as myosin heavy chain genes (*MyHC* genes) and muscle creatine kinase (*M-CK*) are expressed in the last phase of this multi-regulated program, where mononucleated myocytes specifically fuse to each other to form multinucleated myotubes [22–28].

Dicer loss-of function studies clarified the importance of miRNAs in normal skeletal muscle development [29]. miRNAs actively take part in the proliferation and differentiation of skeletal muscle cells as an integral component of genetic regulatory circuitries.

miR-1, miR-133a/b and miR-206 are largely studied and defined muscle-specific miRNAs (myomiRs). They are regulated in muscular transcriptional networks via MRFs and via others key-regulators of the myogenic program, MEF2 (myocyte enhancer factor 2) and SRFs (serum response factors). Recently, a new regulatory pathway, the mechanistic target of rapamycin (mTOR) signaling was seen to regulate miR-1 expression and was also found responsible for MyoD stability [30–36]. It is possible to functionally define miR-133 as enhancer of myoblast proliferation while miR-1 and miR-206 as enhancers of skeletal muscle differentiation [37–40]. An up-to-date list of the identified targets of miR-1, miR-133 and miR-206, together with a plethora of specific muscular pathways they are involved in, is reported in a recent review [40] and some of these will be also discussed in the next paragraph to highlight how these important families of miRNAs contribute to determine typical deficiencies occurring in a pathological muscular context. Intriguingly, these myomiRs have been shown to behave as serum biomarkers in DMD patients. They are released into the bloodstream as a consequence of fiber damage and their power as diagnostic tools is promising since increased miRNA levels correlate with severity of the disease, significantly better than other commonly utilized markers, such as creatine kinase (CK). Moreover, their major serum stability is another aspect that may make them useful not only for diagnosis but also for monitoring the condition of affected individuals after a therapeutic treatment [41,42].

miR-208b/miR-499, also named myomiRs because of their muscle-restricted expression, are produced from the introns of two myosin genes, *β-MHC* and *Myh7b*. They are functionally redundant and play a dominant role in the specification of muscle fiber identity by activating slow and repressing fast myofiber gene programs [43].

Interestingly, many miRNAs are defined as “non-muscle specific” (or also ubiquitously expressed), because essentially they are not exclusively expressed in muscular tissue. It has been, however, demonstrated that they play key-roles in modulating important pathways involved in the regulation of muscular metabolism and cellular commitment. Many miRNAs fall into this category and we report here a few relevant examples, providing for each miRNA the context in which they were studied and highlighting their global effects on muscular metabolism (Table 1).

Some of these miRNAs counteract the differentiation process since their activity is aimed to positively regulate the proliferation phase during muscular development.

miR-125b, one of the few down-regulated miRNAs during myogenesis, together with miR-221/222, negatively contributes to myoblast differentiation and muscle regeneration, taking part in the regulatory axis that includes mTOR and IGF-II [50–52]. Similarly, miR-155 mediates the repression of differentiation targeting MEF2A, a member of MEF2 family of transcription factors. By this negative regulation, miR-155 functions as an important regulator of muscle gene expression and myogenesis [53]. miR-221/222 instead are involved in maintenance of the proliferative state promoting cell cycle progression. They are under control of the Ras-MAPK axis and inhibit the cell-cycle regulator p27 (Cdkn1b/Kip1). Their ectopic expression, indeed, lead to defects in the transition from myoblasts to myocytes and in the assembly of sarcomeres in myotubes [58].

**Figure 1:**
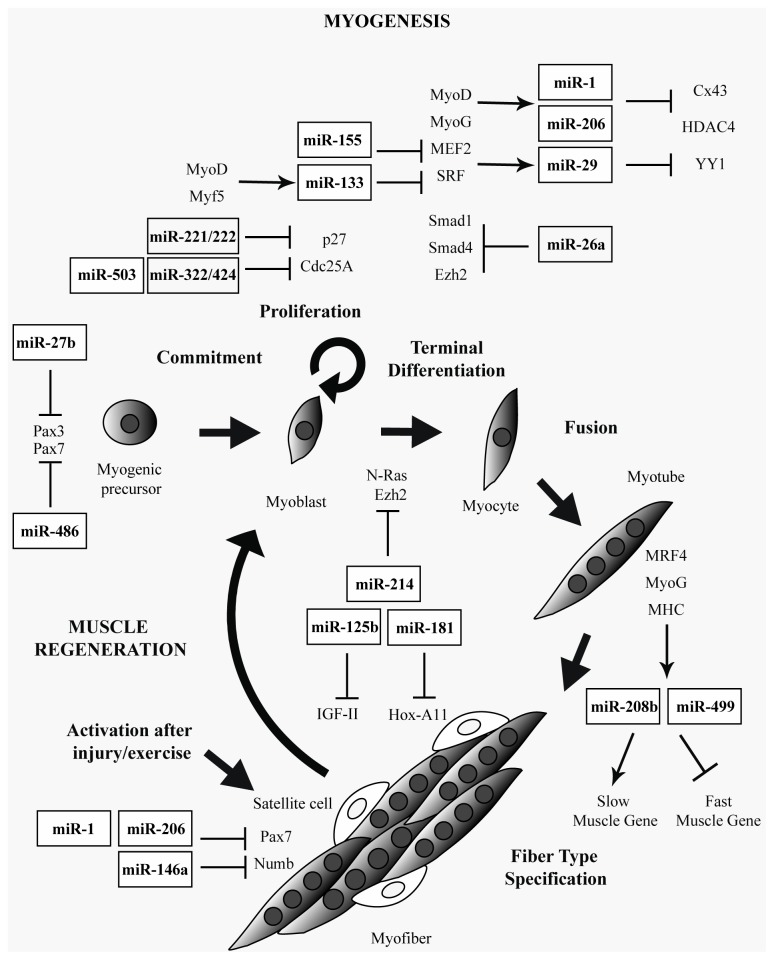
Overview of muscle-specific and ubiquitously expressed miRNAs that contribute to myogenesis and muscle regeneration processes and their regulatory activity on the muscular specific targets/chromatin modifying enzymes/cell cycle regulators (for details see the text). The main regulatory factors that exert a fundamental role during each step of normal muscle development are also reported as well as their eventual regulatory activity on the described miRNAs.

In contrast to this set of miRNAs, many other “non-muscle specific” miRNAs exert an active role in muscle differentiation through different mechanisms: miR-24, for example, has been shown to be essential for the modulation of transforming growth factor β/bone morphogenetic protein (TGF-β/BMP) pathway, a well-known inhibitor of differentiation, although its specific muscular targets are yet unknown [44]; miR-26a is involved in TGF-β/BMP pathway, where it negatively regulates the transcription factors Smad1 and Smad4, critical components of that signaling; miR26a targets the polycomb complex member Ezh2, involved in chromatin silencing of skeletal muscle genes [45,46]; miR-27b promotes entry into differentiation program both *in vitro* and *in vivo* regenerating muscles by down-regulating Pax3 [47]; miR-29 in general is defined as an enhancer of differentiation. During myogenesis it is up-regulated by SRFs and MEF2, and in a self-regulatory manner, it suppresses YY1 and HDAC4 translation by targeting their 3′-UTRs [48,49]; miR-146a is another positive regulator of myogenesis, since it modulates the activity of NUMB protein, which promotes satellite cell differentiation towards muscle cells by inhibiting Notch signaling [55,56]; miR-181 is involved in skeletal muscle differentiation and regeneration after injury and one of its targets is Hox-A11, which in turn represses transcription of MyoD [54]; miR-214 was identified in zebrafish as regulating the muscle development. Here it is expressed in skeletal muscle cell progenitors and was shown to specify muscle cell type during somitogenesis by modulating the response of muscle progenitors to Hedgehog proteins signaling [57]. Its involvement in muscle is also confirmed in C2C12 myoblasts and in skeletal myofibers of mouse where it promotes cell cycle exit and thus differentiation, targeting proto-oncogene N-*Ras* and the repressor of myogenesis Ezh2 respectively [62,63]; miR-322/424 and -503 promote myogenesis interfering with the progression through the cell cycle [59]; while miR-486 was reported to positively regulate myoblast differentiation targeting phosphatase and tensin homolog (PTEN) and Foxo1a, which negatively affect phosphoinositide-3-kinase (PI3K)/Akt signaling and down-regulate the transcription factor Pax7, required only for muscle satellite cell biogenesis and specification of the myogenic precursor lineage [60,61]. All these data clearly show the vast scenario of functions in which miRNAs are involved and their specific activities they play in the skeletal muscle physiology (Figure 1).

## 3. miRNAs in Muscular Dystrophies

Muscle is a dynamic tissue that goes through many recurrent phases of degeneration and regeneration throughout an individual’s lifetime. During normal muscle development, specific molecular circuitries and signaling pathways control several events in different cell types such as activation of satellite cell proliferation, progenitor cell maintenance, myoblast differentiation, muscle cell homeostasis and immune cell recruitment. It is therefore not surprising that their deregulation heavily contributes to the degeneration of dystrophic muscles and is the object of intense research [64] (Table 2).

Eisenberg *et al.* analyzing 10 primary muscular disorders (including DMD, BDM, LGMD and FSHD samples) have identified five miRNAs (miR-146b, miR-221, miR-155, miR-214, and miR-222) consistently deregulated in almost all samples taken into consideration, suggesting their involvement in common regulatory mechanisms. Other miRNAs however showed a disease-specific profile. Functional correlation between miRNAs and mRNA targets in DMD biopsies draw a tight posttranscriptional regulation network in secondary response functions and in muscle regeneration [71].

Greco and coworkers have divided a DMD-signature of miRNAs into three main classes relative to their functional link to specific muscular pathway. Regeneration-miRNAs were up-regulated (miR-31, miR-34c, miR-206, miR-335, miR-449, and miR-494), while degenerative-miRNAs (miR-1, miR-29c, and miR-135a) were down-regulated in *mdx* mice and in DMD patients’ muscles. The third class are named inflammatory-miRNAs, (miR-222 and miR-223), being expressed in damaged muscle areas only [65].

Muscle specific myomiR miR-1 and miR-133 and the ubiquitous miR-29c and miR-30c are down-regulated in *mdx* mice. It is possible to restore WT levels of these miRNAs by treating animals with an exon-skipping approach to restore a partially functional dystrophin protein, an experimental strategy that overcomes an out-frame mutation in the *DMD* locus. The same results are confirmed also in human DMD samples. These results corroborate the direct correlation between miRNAs levels and dystrophin protein levels. In contrast with the other myomiRs, miR-206 shows an increased expression in distrophic *mdx* muscle because it activates satellite cell differentiation program through Pax7 and HDAC4 repression. Another interesting target of miR-206 is Utrophin (Utrn), a dystrophin protein homolog, involved in a compensatory mechanism in DMD pathology [31,66,74].

miR-31-repressing activity seems to regulate muscle terminal differentiation directly targeting the 3′-UTR of dystrophin. Also miR-31, as miR-206, has a preferential localization in regenerating myoblasts, and is highly expressed in Duchenne muscles, probably due to an intensified activation of satellite cells. In both human and murine wild-type conditions its expression is detected in early phases of myoblast differentiation, supporting the idea that it contributes to avoid early expression of late differentiation markers. For this reason it is linked to a delay in the maturation program occurring in the pathological context [70].

**Figure 2:**
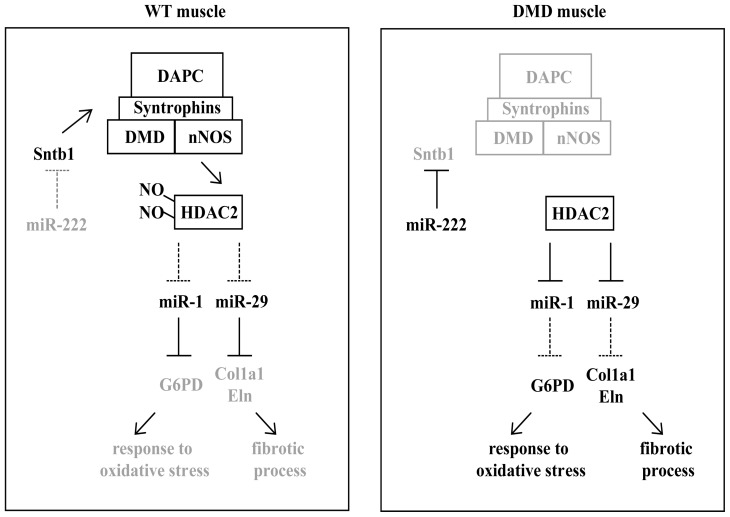
Schematic representation of the functional/physical relationship between Dystrophin-Syntrophins-nNOS pathway and miRNAs involved in such signaling, both in a WT and a DMD context.

Dystrophin is a structural protein that links the cytoskeleton to a large membrane-associated multiprotein complex (dystrophin-associated protein complex, DAPC) to stabilize the sarcolemma. Via Syntrophins (SNTA1, SNTB1, SNTB2, SNTG2), members of DAPC, the enzyme neuronal Nitric Oxide Synthase (nNOS) is localized to the membrane of muscle fibers and regulates intramuscular generation of nitric oxide (NO) [75–77]. nNOS signaling determines the status of nitrosilation of Histone Deacetilases (HDACs) and thus their chromatin association to muscular specific gene-targets. Upon myoblast differentiation, HDACs are displaced from chromatin to promote muscle-specific gene transcriptional activation [78,79]. Some miRNAs involved in DMD pathology have been recently discovered to undergo this type of transcriptional regulation [66]. The absence of dystrophin in DMD patients and *mdx* mice leads to a dramatic decrease of DAPC and a consequential impairment of NO production [80,81]. The expression of a specific subset of miRNAs is modulated by HDAC2 via Dystrophin/nNOS pathway. In particular the activation of both human and murine miR-1 and miR-29 is tightly linked to HDAC2 release from their respective promoters. The functional role of these two miRNAs in muscular metabolism is also been highlighted. miR-1 controls Glucose-6-phosphate dehydrogenase (G6PD), a relevant enzyme involved in the response to oxidative stress while miR-29 controls fibrotic process since it targets the structural component of extracellular matrix, collagen (Col1a1) and elastin (Eln). Moreover, miR-222 targeting β1-Syntrophin (Sntb1) may also contribute to deregulation of the Dystophin-Syntrophins-nNOS pathway [72] (Figure 2).

Myotonic dystrophy (DM) is the most common adult onset, progressive muscular dystrophy. DM is a multi-systemic disease and it is characterized by a generalized muscle weakness and wasting, associated with peripheral neuropathy, heart rhythm defects, and cataracts. The myotonia phenomenon is due to the peculiar muscle membrane depolarization activities. Two type of DM exist, type-1 (DM1, OMIM 160900) and type-2 (DM2, OMIM 602668). DM1 is caused by an expansion of the CTG triplet repeats in the 3′-untraslated region (UTR) of the Dystrophic Myotonic Protein Kinase (*DMPK*), while DM2 is caused by the expansion of a tetranucleotide repeat CCTG in the first intron of CCHC-type zinc finger nucleic acid binding protein (*CNPB*). These gene expansions do not disrupt the relative protein coding sequence, the repeats being in non-coding regions. However, both expanded RNAs accumulate in the nucleus and trigger a toxic gain of function that interferes with RNA splicing of other genes [82–86]. Perbellini and colleagues have performed expression analysis in DM1 biopsies obtained from 15 patients. They found specific deregulated miRNAs: miR-1 and -335 are up-regulated, whereas miR-29b, -29c and -33 are down-regulated compared to control muscles [67,68]. Gambardella and co-workers profiled a specific pattern of myomiRs involved in myogenesis of cardiac and skeletal muscle and found lines of evidence of miR-206 overexpression in five DM1 patients [69]. A similar investigation has been made in DM2 patients. Eleven miRNAs have been shown to be deregulated. Nine displayed higher levels compared to controls (miR-34a-5p, miR-34b-3p, miR-34c-5p, miR-146b-5p, miR-208a, miR-221-3p and miR-381), while four were decreased (miR-125b-5p, miR-193a-3p, miR-193b-3p and miR-378a-3p). Moreover the potential involvement of these miRNAs in relevant skeletal muscle pathways and functions has been validated by bioinformatics analyses [73]. Recently a novel therapeutic approach has been proposed to target the CTG repeat expansion on RNA using antisense oligonucleotides [8,87,88]. Therefore improving knowledge concerning the transcription regulation of the *DMPK* gene, also via ncRNAs, will greatly benefit this new therapy.

## 4. Long Non-Coding RNAs in Skeletal Muscle and Muscular Dystrophies

Increasing lines of evidence support the biological relevance of lncRNAs. They are regulated during development and involved in almost all levels of gene expression and cellular functions including chromosomal dosage compensation, chromatin modification, cell cycle regulation, control of imprinting, alternative splicing, intracellular trafficking, cellular differentiation, and reprogramming of stem cells [89]. Recently, lncRNAs related to muscle are emerging both in physiological and pathological context (Table 3).

Key features of dystrophic muscle include central nuclei, small regenerating fibers and accumulation of connective tissue and fatty tissue. Muscle differentiation *in vitro* is a useful system to investigate the activity of long non-coding RNAs that show muscular specific pattern of expression. Recently, a new regulatory network involving cross-talk of several ncRNAs has been identified by Cesana and colleagues. Relying on ability of myomiRs to orchestrate muscular proliferation and differentiation, the genomic region of miR-206/-133b has been analyzed in detail. Thus a novel muscle specific transcript has been identified. Because of its non-coding potential and its activated expression upon myoblast differentiation it was termed linc-MD1. More specifically linc-MD1 is expressed in newly regenerating fibers and is abundant in dystrophic condition, however no expression is detected in mature differentiated fibers. linc-MD1 is localized in the cytoplasm and is a polyadenylated transcript. Through a series of functional studies it was possible to define its competing endogenous activity (ceRNA). linc-MD1 acts as a natural decoy for miR-133 and -135, thus interfering with miRNA repressing activity on the important targets involved in myogenic differentiation MAML1 (Mastermind-like 1) and MEF2, respectively [90].

Metastasis associated lung adenocarcinoma transcript 1 (Malat1) is a highly conserved 8.7 kb non-coding transcript that is abundantly expressed in cancer cells and a strong predictor of metastasis [102]. Malat1 has been proposed to regulate alternative splicing [103], transcriptional activation and the expression of nearby genes [104,105]. Numerous experimental examples support its functional role in the regulation of cell growth, but the exact mechanism of action of Malat1 in different physiological and pathological conditions still needs to be elucidated. By a microarray data analysis obtained using skeletal muscle of mice (gastrocnemius muscle) treated with recombinant myostatin it was observed that the Malat1 expression levels are significantly decreased. Myostatin is a potent negative regulator of myogenesis that inhibits myoblast proliferation and differentiation [106,107]. Further expression analysis confirmed a persistent up-regulation of Malat1 during the differentiation of myoblasts into myotubes in C2C12 cells as well as in primary human skeletal muscle cells. Conversely, targeted knockdown of Malat1 using siRNA suppressed myoblast proliferation by arresting cell growth in the G0/G1 phase. These results reveal Malat1 as a novel downstream target of myostatin with a considerable ability to regulate myogenesis. Although Malat1 appears largely dispensable for normal mouse development [108,109] it is plausible that Malat1 has a role in the transition from the proliferative phase to differentiation in skeletal myogenesis, as well as in the commitment to muscle differentiation [91].

Many lncRNA have been discovered but not yet fully characterized, as for example Men ɛ/β lncRNAs. To date it is known that two long non-coding isoforms (Men ɛ/β lncRNAs) which are expressed in several human tissues, including muscle, arise from the Multiple Endocrine Neoplasia I locus (*MEN1*). Experimental lines of evidence show their up-regulation upon differentiation of C2C12 myoblats, although their biological role in muscular development is not yet clear. Men ɛ (also known as NEAT1) and Men β are transcribed from the same RNA polymerase II promoter and are both retained in the nucleus. Suwoo and colleagues formally demonstrated that Men ɛ/β transcripts are critical structural/organizational components of paraspeckles, organelles localized in the nucleoplasm close to nuclear speckles, where RNA-binding proteins and *Cat2*-transcribed nuclear RNA (CTN-RNA) are stored [110]. Moreover, large-scale analysis revealed that many other lncRNAs are differentially expressed in C2C12 cells upon myoblast differentiation into myotubes, although their biological functions have not been investigated [92–94].

Between the many functions ascribed to lncRNA there are examples of lncRNAs modulating the activity of transcriptional activators or co-activators, directly or through the regulation of their sub-cellular localization [89]. Two of these have been seen also in a muscular context. The steroid receptor RNA activator (SRA) RNA is a very peculiar transcript that exists as both a non-coding and a coding RNA (yielding SRA ncRNA and protein SRAP respectively). The SRA ncRNA is highly expressed in skeletal muscle and works as a co-activator of MYOD transcription factor, a master regulator of skeletal myogenesis. To address the significance of the enigmatic bifunctional property of this transcript, Hube and colleagues performed an exhaustive analysis clarifying the opposite function of non-protein coding SRA versus ORF-containing transcripts. The balance between coding and non-coding SRA isoforms changes during myogenic differentiation in primary human cells. In particular it is shown that an increased expression of SRA ncRNA and a parallel decrease of protein SRAP occurs during myogenic differentiation in healthy muscle satellite cells. This does not happen in cells isolated from DM1 patients, probably because of a delay in differentiation program. Remarkably, only the ncRNA species enhances MYOD transcriptional activity. The protein SRAP prevents this SRA RNA-dependent co-activation through interaction with its RNA counterpart [95–97]. However how this is achieved is not known.

Non-coding repressor of NFAT (NRON) is another case of lncRNA that shows a regulatory activity on a transcription factor. NRON is not highly expressed but it has a distinct tissue specific expression. It has been found enriched in placenta, muscle, and lymphoid tissues. NFAT is a transcription factor responsive to local changes in calcium signals. It is essential for the T cell receptor–mediated immune response and plays a critical role in the development of heart and vasculature, musculature, and nervous tissue. The first study about the role of NRON showed that it regulates NFAT’s subcellular localization rather than its transcriptional activity. Sharma and coworkers confirmed these data demonstrating that NRON takes part in a large cytoplasmic RNA-protein complex that acts as a scaffold for NFAT to modulate its nuclear trafficking and thus its response activity [98,99].

Little is yet known about the dystrophin gene regulation. *DMD* is the largest gene in the human genome that comprises 79 exons spanning >2500 kb on chromosome Xp21.2, which gives rise to 7 isoforms that are finely regulated in terms of tissue specificity [111]. Mutations in the *DMD* gene range from single-nucleotide changes to chromosomal abnormalities (http://www.dmd.nl/). Deletions encompassing one or more exons of the dystrophin gene are the most common cause of the severe Duchenne muscular dystrophy (DMD) resulting in an absence of dystrophin or expression of a non-functional protein. Becker muscular dystrophy (BMD) instead is a milder form of dystrophy because it is associated with reduction of wild-type dystrophin or expression of a partially functional protein. DMD is the most common inherited muscle disease affecting approximately one in 3500 males and is characterized by progressive muscle wasting during childhood. Heterozygous females for dystrophin mutations are named carriers of DMD mutations [112,113]. Many of them are asymptomatic, but a certain number, defined as “manifesting” or “symptomatic”, develop symptoms of the disease, which vary from a mild muscle weakness to a DMD-like clinical course. Despite intensively explored, the pathogenic mechanism underlying clinical manifestation in DMD female carriers still remains a controversial issue [114]. For these reasons *DMD* regulation is a field of intense interest to shed light on this complex scenario.

Using a custom-made tiling array the entire *DMD* gene has been explored in the search for non-coding transcripts originating within the dystrophin locus. The major tissues of dystrophin synthesis, namely human brain, heart and skeletal muscle, were used as test tissues in array. The data analysis has highlighted a variety of novel long non-coding RNAs (lncRNAs), both sense and antisense oriented, whose expression profiles mirror that of *DMD* gene. Importantly, these transcripts are intronic in origin, specifically localized to the nucleus and are transcribed contextually with dystrophin isoforms or in fibroblast upon MYOD-induced myogenic differentiation. To characterize their possible functional role on the *DMD* locus three sense-oriented lncRNAs (lncINT44s, lncINT44s2 and lncINT55s) isolated from skeletal muscle were further investigated. Their forced ectopic expression in both human muscle and neuronal cells causes a negative regulation of endogenous full-length dystrophin isoforms, denoted B for brain (Dp427b), M for muscle (Dp427m) and P for Purkinje (Dp427p). Importantly, no variation was observed with regard to the ubiquitous Dp71 transcript, suggesting that the effect of sense lncRNAs on full-length dystrophin isoforms may be specific. In particular, reporter assay confirmed their repressive role on the minimal promoter regions of the muscle dystrophin isoform. A possible mechanism of action involves specific DMD lncRNAs that control muscle dystrophin isoforms by down-modulating dystrophin transcription levels. An inverse correlation between ncRNAs expression and muscle dystrophin has been also found *in vivo*, analyzing muscle samples of DMD female carriers, either healthy or mildly affected, reinforcing the idea that a negative relationship between lncRNAs and dystrophin mRNA levels may exist [14].

In severe DMD one third of patients display also mental retardation, but the pathogenesis is unknown. In a singular case of DMD complicated by mental retardation, an intra-chromosomal inversion (inv(X)p21.2;q28) has been identified. The genetic rearrangement has been molecularly characterized to find a possible disrupted gene because of the inversion, and that might be responsible for the neurological symptoms associated with dystrophy. A novel gene named *KUCG1* was discovered at break point on Xq28. The 658-bp transcript displays an mRNA-like structure but not having coding potential is been classified as long non-coding RNA. KUCG1 lncRNA is expressed at low levels in a tissue-specific manner, as well in the brain. It is possible that the disruption of KUCG1 transcript contributes to the development of mental retardation in the index case [115] since other experimental lines of evidence suggest that a subset of lncRNAs could contribute to neurological disorders when they become deregulated [100].

Polycomb (PcG) and Trithorax (TrxG) group proteins antagonistically act in the epigenetic regulation of gene expression. Typically, TrxG counteracts PcG-mediated epigenetic gene silencing. Among the many lncRNAs interacting with chromatin remodeling enzymes the most famous are Xist and HOTAIR, both acting as a negative regulators of gene expression by recruitment of PRC2 (Polycomb Repressive Complex 2) on PcG target genes [116,117]. Cabianca *et al*. were the first to discover an lncRNA interacting with the TrxG in the Facioscapulohumeral muscular dystrophy (FSHD). FSHD is an autosomal-dominant disease characterized by progressive wasting of facial, upper arm, and shoulder girdle muscles. In up to 95% of cases, the genetic defect is mapped to the subtelomeric region of chromosome 4q35 containing a macrosatellite tandem array of 3.3 Kb long D4Z4 repeats. FSHD is caused by deletions reducing copy number of D4Z4 below 11 units rather than a classical mutation in a coding-protein gene. D4Z4 deletion is associated to a loss of repressive epigenetic marks and thus to a switch from a heterochromatic/close state to a more euchromatic/open conformation of chromatin structure. A novel long non-coding RNA, named DBT-E is produced selectively in FSHD patients. DBT-E is transcribed from D4Z4 repeats and is a chromatin-associated lncRNA that coordinates de-repression of genes located in the 4q35 region. DBT-E recruits the Trithorax group protein Ash1L to the FSHD locus driving histone H3 lysine dimethylation and thus chromatin remodeling [101].

## 5. Discussion

It is surprising how ncRNAs are tightly interconnected with the main fundamental aspects of muscular tissue: development, differentiation and regeneration. At the molecular level miRNAs and lncRNAs take part in almost all levels of regulation in these key processes. Chromatin modifying enzymes, positive and negative transcription factors, cell cycle regulators, and enzymatic and structural proteins involved in signaling circuitries are under their fine-modulation. Moreover, ncRNAs are often found to be under the regulation of their own targets, thus determining feedback loops that drive developmental switches ensuring a perfect synergy between stimuli and responses. Both time- and tissue-specific gene regulation are the fulcrum on which the fine-tuning of a healthy organism is based. Disrupting the physiological pattern of expression not only in codifying genes, but also in regulatory RNAs, can heavily modify specific cell processes.

If this is true in physiological conditions, increased lines of evidence show that regulatory RNAs play a crucial role also in the etiology of many human diseases. Among these, muscular dystrophies represent a field of intense research, also because of the recent creation of novel experimental treatments. This has encouraged studies on expression regulation in diseases muscle cells, both *in vivo* (animal models) and *in vitro*. These studies have shown that mutant proteins in MDs result in perturbations of many cellular components. Indeed MDs have been associated with mutations in structural proteins, signaling molecules and enzymes as well as mutations that result in aberrant processing of mRNA or alterations in post-translational modifications of proteins. These findings have not only revealed important insights for cell biologists, but have also provided unexpected and exciting new approaches for therapy. Moreover, in muscular dystrophies as well as in other diseases, such as cancer, regulatory RNAs may serve as biomarkers, providing information on disease course, disease severity and response to therapies. miRNA dosing in serum is a very appealing field of investigation since they are easily accessible, peculiar to defined conditions and can facilitate the early identification of the muscular disease, potentially avoiding invasive techniques such as a biopsy, or in some cases to reduce the time and the costs of diagnosis. Biomarkers are particularly important in the field of personalized treatments. Pharmacogenomics aims at predicting which drug will be most effective and safe in the individuals. This can be established via genome sequence and SNP association (pharmacogenetics) and expression profiling. miRNAs have been shown to play a pivotal role in drug efficacy and toxicity, having powerful implications in personalized medicine [118]. Indeed, as we have described, miRNAs can negatively regulate gene expression and can profile the disease severity, as in the case of myomiRs and DMD [41]. miRNAs show a linear relationship with genes and drugs, since drug function can be influenced or even hampered by changes in genes expression level or in specific isoforms representation, as supported by several data on cancer [119]. Many pharmacogenomically relevant genes are regulated by miRNAs, as summarized and shown in the Pharmacogenomcis Knowledge Base (PharmGKB, www.pharmgkb.org/), a very useful resource listing genes known to be relevant for drug response.

Conversely, miRNAs can vary in their expression level following drug treatments [120]. Within the muscle field, we do have increased knowledge on the miRNAs network, especially those governing the muscle transcriptional network. It is clearly emerging how miRNAs can regulate differentiation and homeostasis of skeletal muscle progenitor cells, providing robustness to the MYOD-induced myoblast differentiation and myogenesis [121,122]. Disclosing the role miRNAs have in regulating the intermediate steps of the myogenesis cascade will be of outmost importance in identifying drugs that may act as adjuvants/enhancers of gene/protein re-synthesis in clinical trials, as for exon skipping therapies in DMD.

More complex is our current understanding of the role of lncRNAs in muscle biology and pathology. We have just started to explore the peripheral areas of this “*terra incognita*”. So far lncRNAs have been involved in numerous molecular processes such as remodelling of chromatin architecture, or regulation of gene transcription. For instance, some pharmacodynamic studies on corticosteroids, which represent the gold standard in the routine therapy of DMD, revealed that the steroid receptor RNA activator (SRA) transcript functions as both a lncRNA and template for synthesis of a protein (SRAP). Interestingly, the SRA ncRNA increases the activity of nuclear receptors (not only for corticosteroids) and acts as a master regulator of MYOD expression [95]. lncRNAs can also exert their function through a more passive role. For instance they are valued for their ability to work as molecular sponges by annealing to small RNAs and thereby preventing them from their normal activity. Furthermore, in some other cases lncRNAs have been shown to provide a kind of structural backbone for the assembly of ribonucleic particles whose functions are still to be disclosed. In this respect, it has been crucial to determine in which intracellular compartments these RNA/protein particles form. Despite the fact that so far most investigated lncRNAs are confined to nuclei, a few recent studies have, indeed, shown that some lncRNAs can also abundantly localize inside the cytoplasm with functions that still remain to be determined.

Given that just a few lncRNAs have been tackled on functional levels and that the annotated ones are in the order of thousands with many more expected to be discovered, it is plausible to speculate that their involvement in novel functions and roles will be rapidly identified with repercussions on many fields of cell biology and pathology and with the possibility to potentially employ them as biological markers as well as drugs to treat major diseases such as muscular dystrophies.

## 6. Conclusions

Although in the majority of cases the etiology of muscular dystrophies is not ascribed to functional non-coding RNA molecules (with the exception of FSHD), they appear as powerful regulators of several key-pathways and show how actively they can contribute to the progression of disease. This reflects the strong ability of miRNAs and lncRNAs in the modulation of the phenotype of dystrophic affected individuals via fine regulatory pathways that can lead to increased transcript stability, mRNA splicing control, enhanced protein production, posttranslational protein modification and other mechanisms. These versatile roles support the idea to use regulatory RNAs as novel targeted molecules acting as enhancers or inhibitors in well-established therapeutic strategies (based both on drugs and gene therapies). After all, the general goal is to ameliorate the final output of the specific treatments.

## Figures and Tables

**Table 1:** miRNAs expressed in muscular tissue (in an exclusive manner or not) and their global effect on muscle metabolism.

miRNA	Role in Muscle Metabolism [Refs.]	Tissue Expression
miR-1	enhancer of skeletal muscle differentiation [37–40]	muscle-specific
miR-133a/b	enhancer of myoblast proliferation [37–40]	muscle-specific
miR-206	enhancer of skeletal muscle differentiation [37–40]	muscle-specific
miR-208b	involved in specification of muscle fiber identity [43]	muscle-specific
miR-499	involved in specification of muscle fiber identity [43]	muscle-specific
miR-24	promotes myoblast differentiation [44]	ubiquitous
miR-26a	promotes myoblast differentiation [45,46]	ubiquitous
miR-27b	promotes entry into differentiation program [47]	ubiquitous
miR-29	enhancer of differentiation [48,49]	ubiquitous
miR-125b	negatively contributes to the myoblast differentiation and muscle regeneration [50–52]	ubiquitous
miR-155	represses myoblast differentiation [53]	ubiquitous
miR-181	regulates skeletal muscle differentiation and regeneration after injury [54]	ubiquitous
miR-146a	promotes satellite cell differentiation [55,56]	ubiquitous
miR-214	promotes cell cycle exit and differentiation [57]	ubiquitous
miR-221/222	promote cell cycle progression [58]	ubiquitous
miR-322/424; miR-503	promote myogenesis interfering with the progression through the cell cycle [59]	ubiquitous
miR-486	positively regulates myoblast differentiation [60,61]	muscle-enriched

**Table 2:** miRNAs found deregulated in MDs and their specific activity on muscular targets or involvement in muscular processes.

miRNA/miRNAs	Deregulated in MDs [References]	Type of Deregulation	Muscular Targets/Process [References]
miR-1 (myomiR)	DMD [65,66]; DM1 [67,68]	down-regulated	HDAC4; Cx43; Pax7; c-Met; G6PD [40]
miR-133 (myomiR)	DMD [66]	down-regulated	SRF; nPTB; UCP2 [40]
miR-206 (myomiR)	DMD [65]; DM1 [69]	up-regulated	DNApolα; Fstl1; Utrn; Pax7; Cx43; HDAC4; c-Met [40]
miR-29b/c	DMD [65,66]; DM1 [67]	down-regulated	YY1; Col1a1; Eln; HDAC4 [40,62,63]
miR-135a	DMD [65]	down-regulated	muscle degeneration [65]
miR-30c	DMD [66]	down-regulated	-
miR-31	DMD [65,70]	up-regulated	DMD [70]
miR-34c; miR-449; miR-494	DMD [65]	up-regulated	muscle regeneration [65]
miR-146b; miR-155	DMD; BMD; LGMD; FSHD [71]	up-regulated	-; MEF2A [53]
miR-214	DMD; BMD; LGMD; FSHD [71]	up-regulated	Ezh2; N-Ras [40,62,63]
miR-221; miR-222	DMD; BDM; LGMD; FSHD [71]	up-regulated	p27(Cdkn1b/Kip1) [58]; Sntb1 [72]
miR-223	DMD [65]	up-regulated	muscle inflammation [65]
miR-335	DMD [65]; DM1 [67]	up-regulated	muscle regeneration [65]
miR-33	DM1 [67]	down-regulated	-
miR-34a-5p; miR-34b-3p; miR-34c-5p; miR-146b-5p; miR-208a; miR-221-3p; miR-381	DM2 [73]	up-regulated	-
miR-125b-5p; miR-193a-3p; miR-193b-3p; miR-378a-3p	DM2 [73]	down-regulated	-

**Table 3:** Recently discovered lncRNAs related to muscle, both in physiological and pathological context.

lncRNA/lncRNAs [References]	Expression in Muscular Districts	Deregulated in MDs	Activity
linc-MD1 [90]	Expressed in newly regenerating fibers	DMD	natural decoy for miR-133 and -135 (ceRNA)
Malat1 [91]	up-regulated during the differentiation of myoblasts into myotubes	?	regulation of cell growth
Men ɛ/β lncRNAs [92–94]	up-regulated upon differentiation of C2C12 myoblats	?	critical structural/organizational components of paraspeckles
SRA ncRNA [95–97]	increased expression during myogenic differentiation	DM1	co-activator of MYOD transcription factor
NRON [98,99]	enriched also in muscle	?	regulates NFAT’s subcellular localization (scaffold)
lncINT44s; lncINT44s2; lncINT55s [14]	transcribed contextually with dystrophin isoforms and upon MYOD-induced myogenic differentiation	?	negative modulation of endogenous dystrophin full-length isoforms
KUCG1 [100]	expressed at low levels in the brain	DMD with mental retardation	possible candidate gene that contribute to develop of mental retardation in the index case
DBT-E [101]	not-physiological lncRNA	FSHD	coordinates de-repression of genes located in the 4q35 region
